# HF etching of CAD/CAM materials: influence of HF concentration and etching time on shear bond strength

**DOI:** 10.1186/s13005-019-0206-8

**Published:** 2019-08-08

**Authors:** Antonio Straface, Lena Rupp, Aiste Gintaute, Jens Fischer, Nicola U. Zitzmann, Nadja Rohr

**Affiliations:** Department of Reconstructive Dentistry, University Center for Dental Medicine, University of Basel, Mattenstrasse 40, CH-4058 Basel, Switzerland

**Keywords:** Shear bond strength, CAD/CAM, Ceramic, HF etching time, HF concentration

## Abstract

**Background:**

The required pretreatment of CAD/CAM ceramic materials before resin composite cement application varies among studies. The aim of the present study was to evaluate the effect of hydrofluoric acid concentration and etching time on the shear bond strength (SBS) of two adhesive and two self-adhesive resin composite cements to different CAD/CAM ceramic materials.

**Methods:**

SBS of two adhesive (Panavia V5, Kuraray, [PV5]; Vita Adiva F-Cem, Vita Zahnfabrik, [VAF]) and two self-adhesive (RelyX Unicem 2 Automix, 3 M Espe, [RUN]; Vita Adiva S-Cem, Vita, [VAS]) cements to four different CAD/CAM materials (Vitablocs Mark II, Vita, [VM]; Vita Enamic, Vita, [VE]; e.max CAD, Ivoclar Vivadent, [EC]; Vita Suprinity PC, Vita, [VS]) was measured. The effect of the surface pretreatment by using two different hydrofluoric acid products (HF5% Vita Ceramics Etch, Vita and HF9% buffered, Ultradent Porcelain Etch, Ultradent Products) were assessed at etching times of 0 s, 5 s, 15 s, 30s and 60s for each cement and restorative material combination (*n* = 10 per group, total *n* = 1440).

**Results:**

Significant effects were found for the etching time and cement for all materials with highest shear bond strength for etching times of 60s = 30s = 15 s ≥ 5 s > 0 s and for RUN>PV5 = VAF > VAS (*p* < 0.05). Etching with HF5% for 5 s to 15 s resulted in higher SBS values, while no differences were observed between HF5% and HF9% buffered when the substrates were etched for 30s to 60s (*p* < 0.05).

**Conclusions:**

Within the limitations of this study the recommended surface pretreatment of silicate ceramics is HF etching with concentrations of 5% or 9% for 15 s to 60s to achieve highest shear bond strength while the glassy matrix is sufficiently dissolved. The tested resin composite cements can be applied with all tested materials and suggested for clinical application.

## Background

Digital technology in dentistry was introduced in the late 80s and ever since was constantly developed [1–3]. Together with progressive changes in the equipment industry, new developments of different restorative materials resulted in major breakthroughs of computer-aided-design/computer-aided-manufacturing (CAD/CAM) technologies. Nowadays, computerized manufacturing is often routinely involved in restorative dentistry and is associated with high accuracy, accelerated production speed and reduced manual application [4]. Tooth-colored CAD/CAM restorative materials have been successfully documented over the last two decades with promising performance [5–8].

Feldspar ceramic has been considered a gold standard due to its tooth-like appearance, based on light transmission and natural effect [9, 10]. However, the low mechanical properties, as for instance fracture strength [11–13] was a limiting factor for its application and stimulated further advancements of glass ceramics reinforced with different fillers [14, 15]. Hence, lithium disilicate (LiS2) ceramic (as a high-strength and highly esthetic material) [16] and polymer-infiltrated ceramic (due to similar hardness and elastic modulus to dental structures; higher fracture toughness and reduced brittleness) [17, 18] were developed [9]. The reported survival rate of CAD/CAM-fabricated polymer-infiltrated ceramic inlays is 97.4 and 95.6% for partial coverage restorations after three years [19]. In comparison, the mean survival rate of CAD/CAM LiS2 veneers reaches 99% with 96.4% success after 5 years [20]. However, the high clinical survival and success rates of CAD/CAM single restorations is based not only on the novel materials, but is strongly determined by the strength and durability of the bond formed between the restorative material, luting cement and substrate [9, 21]. Based on current evidence, adhesive bonding of ceramic restorations provides high retention, improves marginal adaptation, prevents micro-leakage and increases fracture resistance of restored teeth and the respective restorations [22–25]. Moreover, resin composite cements are available in tooth-colored shades, which is crucial for luting minimally invasive ceramic restorations [26].

To achieve micromechanical interlocking with the resin composite cement, the surface of silicate ceramics has to be roughened [27–30]. The recommended procedure comprises etching with 5% hydrofluoric acid (HF) and application of silane coupling agent to additionally achieve a chemical bond [31–36]. The application of HF acid reacts with silicate, which leads to the removal of the glass phase and results in an increased ceramic surface area [37–39]. Despite the fact that HF acid is the undisputed ceramic surface etchant, the concentration and etching time are highly controversial, i.e. varying from 5 to 10% and from 15 s to 90s [39–46]. Nevertheless, after the acid etching procedure, the application of silane as a coupling agent linking the hydrophilic restoration surface with hydrophobic resin composite cement is generally recommended [43, 47–49].

In order to simplify the technique-sensitive surface pretreatment for an application of adhesive resin composite cement, self-adhesive cements were introduced [50]. It has been demonstrated that self-adhesive cements are suitable for bonding to dentin, while bonding to enamel substrate is considered inferior in comparison with the etch-and-rinse or self-etch adhesive techniques, in which the applied primer allows further micromechanical interlocking [5, 51–54]. Nonetheless, data on bonding behavior between CAD/CAM ceramic materials, different adhesive cements and surface pretreatment is controversial [32, 34, 49]. Resin composite cements are brittle materials and therefore susceptible to tensile loading rather than to compressive stress [55–57]. Since adhesion of cements to ceramics is commonly tested using a shear bond strength test design, it would be of interest to analyze the effect of the cement’s diametral tensile strength on shear bond strength.

The aim of the present study was to evaluate the effect of hydrofluoric acid concentration and etching time on the shear bond strength (SBS) of adhesive and self-adhesive resin composite cements to different CAD/CAM ceramic materials and to determine the diametral tensile strengths of four different resin composite cements. The hypotheses of the present study were that i) different HF acid concentrations affect the surface morphology of CAD/CAM ceramic materials and shear bond strength, ii) varying HF acid etching times affect the surface morphology of CAD/CAM ceramic materials and shear bond strength.

## Methods

**Fig. 1:**
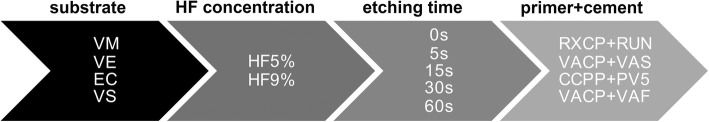
Groups for shear bond strength test. The CAD/CAM materials VM, VE, EC and VS served as substrates for the shear bond strength test. Their surfaces were pre-treated with hydrofluoric acid (HF) of different concentrations and etching times. Afterwards, the respective system primer (RXCP, VACP, CCPP, VACP) and cement (RUN, VAS, PV5, VAF) was applied and shear bond strength was measured after 24 h water storage at 37 °C (*n* = 10 per group)

Shear bond strength (SBS) [30, 33, 50, 58, 59] of two adhesive (Panavia V5 [PV5], Kuraray Noritake; Vita Adiva F-Cem [VAF], Vita) and two self-adhesive (RelyX Unicem 2 Automix [RUN], 3 M Espe; Vita Adiva S-Cem [VAS], Vita) cements to four different CAD/CAM materials (Vitablocs Mark II [VM], Vita; Vita Enamic [VE], Vita; e.max CAD [EC], Ivoclar Vivadent; Vita Suprinity PC [VS], Vita) was measured (Table 1). The effect of the surface pretreatment using two different hydrofluoric acid products (HF5% Vita Ceramics Etch [HF5], Vita and HF9% buffered [HF9], Ultradent Porcelain Etch, Ultradent Products) were assessed at etching times of 0 s, 5 s, 15 s, 30s and 60s for each cement and restorative material combination (Fig. 1, *n* = 10 per group, total *n* = 1440).

**Table 1:** Investigated materials with composition as provided in the safety data sheets of the products

Material	Name	Code	Manufaturer	Type	Composition
Ceramic	Vitablocs Mark II	VM	VITA Zahnfabrik, Bad Säckingen, Germany	Feldspar ceramic	SiO_2_ 56–64%, Al_2_O_3_ 20–23%, Na_2_O 6–9%, K_2_O 6–8% by weight
	Vita Enamic	VE	VITA Zahnfabrik, Bad Säckingen, Germany	Polymer-infiltrated ceramic	86% feldspar ceramic: SiO_2_ 58–63%, Al_2_O_3_ 20–23%, Na_2_O 9–11%, K_2_O 4–6% by weight 14% polymer by weight: Triethyleneglycol dimethacrylate (TEGDMA) / Urethandimethacrylate (UDMA)
	IPS e.max CAD	EC	Ivoclar Vivadent AG, Schaan, Liechtenstein	Lithium disilicate ceramic	SiO_2_ 57–80%, Li_2_O 11–19%, K_2_O 0–13% by weight
	VITA Suprinity	VS	VITA Zahnfabrik, Bad Säckingen, Germany	Zirconia reinforced lithium silicate ceramic	Zirconium oxide 8–12, silicon dioxide 56–64%, lithium oxide 15–21%, various > 10% by weight
Resin composite cement	RelyX Unicem 2 Automix	RUN	3 M ESPE, Seefeld, Germany	Self-adhesive cement	Base Paste: Methacrylate monomers containing phosphoric acid groups, Methacrylate monomers, Silanated fillers, Initiator componenets, Stabilizers, Rheological additives Catalyste Paste: Methacrylate monomers, Alkaline (basic) fillers, Silanated fillers, Initiator components, Stabilizers, Pigments, Rheologicam additives
	VITA Adiva S-Cem	VAS	VITA Zahnfabrik, Bad Säckingen, Germany	Self-adhesive cement	Mixture of dimethacrylates, Glass powder, Fumed silica, Phosphoric esters, Catalysts, Stabilizer, Pigments, Methacrylates, Phosphoric ester
	Panavia V5	PV5	Kuraray Europe GmbH, Hattersheim, Germany	Adhesive cement	Bisphenol A diglycidylmethacrylate (Bis-GMA), Triethyleneglycol dimethacrylate (TEGDMA), Hydrophobic aromatic dimethacrylate, Hydrophilic aliphatic dimethacrylate, Initiators, Accelerators, Silanated barium glass filler, Silanated fluoroalminosilicate glass filler, Colloidal silica Bisphenol A, diglycidylmethacrylate (Bis-GMA), Hydrophobic aromatic dimethacrylate, Hydrophilic aliphatic dimethacrylate, Silanated barium glass filler, Silanated alminium oxide filler, Accelerators, dl-Camphorquinone, Pigments
	VITA Adiva F-Cem	VAF	VITA Zahnfabrik, Bad Säckingen, Germany	Adhesive cement	Mixture of resin based on Bis-GMA, catalyst, stabilizer, pigments, Methacrylates
Ceramic Primer	CLEARFIL CERAMIC PRIMER +	CCPP	Kuraray Europe GmbH, Hattersheim, Germany	Silane + MDP	3-Methacryloxypropyl trimethoxysilane, 10-Methacryloyloxydecyl dihydrogen phosphate (MDP), Ethanol
	VITA ADIVA C-PRIME	VACP	VITA Zahnfabrik, Bad Säckingen, Germany	Silane	Solution of methacrylsilanes in ethanol
	RelyX Ceramic Primer	RXCP	3 M ESPE, Seefeld, Germany	Silane	Ethyl alcohol, Water, Methacryloxypropyltrimethoxysilane
Etching agent	VITA CERAMICS ETCH	HF5	VITA Zahnfabrik, Bad Säckingen, Germany	Hydrofluoric acid	Hydrofluoric acid 5%
	Ultradent Porcelain Etch	HF9	Ultradent Products, Inc., Köln, Germany	Hydrofluoric acid	Hydrofluoric acid 9% buffered

CAD/CAM blocks of each material were cut in slices with a thickness of 3.5 mm using a diamond band saw (Exakt 30–700, Exakt; Norderstedt, Germany) under permanent water-cooling. The substrate slices were then grinded (SiC paper grit P180, Struers, Baltrup, Denmark) to attain a similar roughness as it is given by a CAD/CAM milling machine (Ra = 1.88 μm for VM and VE, 2.71 μm for EC, 2.52 μm for VS after crystallization) [60]. Specimens of EC and VS were additionally crystallized (Vacumat 4000, Vita) according to the recommendation of the manufacturers. For EC temperature increase was 30 °C/min for 15 min up to the crystallizing temperature of 850 °C which was held for 10 min. Cooling temperature was 680 °C. For VS temperature increase was 55 °C/min to 840 °C for 8 min with cooling at 680 °C.

Substrates were cleaned in 70% ethanol prior to crystallizing and before pre-treatment for SBS in an ultrasonic bath (TPC-15, Telsonic, Bronschhofen, Switzerland) for 4 min. Roughness of the specimens was measured with a tactile stylus (Hommeltester T1000, cantilever Type TKK 50, Zug, Switzerland): VM (1.9 ± 0.5 μm), VE (1.8 ± 0.8 μm), EC (0.5 ± 0.1 μm) and VS (0.8 ± 0.1 μm). Hydrofluoric acid etching was performed for either 0 s, 5 s, 15 s, 30s or 60s with HF5 or HF9 and then rinsed thoroughly with water for 20s. After etching, the substrate surfaces were dried with oil-free air and pre-treated with the appropriate ceramic primer recommended by the manufacturer of the respective cement (Table 1, Fig. 1). The primer used in combination with PV5 was Clearfil Ceramic Primer plus [CCPP] (Kuraray Noritake). For both VAF and VAS Vita Adiva Ceramic Primer [VACP] (Vita) was used and to RUN RelyX Ceramic Primer [RXCP] (3 M Espe) was assigned. The respective primers were applied on the substrate surfaces with a microbrush for 20s and dried with oil-free air.

An acrylic cylinder with an inner diameter of 2.9 mm, outer diameter of 4.1 mm, and height of 4 mm was tightened in a custom made device onto the substrate surface to avoid leaking of the cement. The respective cement was filled into the cylinder opening. A steel screw (BN 617, Bossard; Zug, Switzerland) with a diameter matching the inner diameter of the acrylic cylinder was inserted parallel to the axis of the cylinder and loaded with 10 N. The cement was light cured from three different directions for 20s per side (Elipar DeepCure S, 3 M Espe, Neuss, Germany). All specimens were stored in distilled water at 37 °C for 24 h. SBS was measured in a universal testing machine (Z020 Zwick/Roell, Ulm, Germany). The specimens were positioned in the sample holder with the bonding surface parallel to the loading piston. The loading piston had a chisel configuration and was positioned with a distance of 2 mm to the specimen surface. The distance of 2 mm was chosen to prevent extensive cohesive failures by increasing the leverage effect [30, 33]. The load was applied to the outer surface of the cylinder with a crosshead speed of 1 mm/min. Load at failure was recorded, and SBS (σ) was calculated with the following formula: σ = F/πr^2^, in which F is the load in N at fracture and r is the radius of the bonded area of the cylinder in mm (1.45 mm). SBS of specimens that de-bonded during water storage was recorded as 0.0 MPa and included in the statistical analysis. Failure patterns were classified visually as either cohesive failure in the substrate, adhesive failure, mixed or cohesive failure in the cement. Images of those typical failure patterns were obtained with scanning electron microscopy (ESEM XL30, Philips, Eindhoven, the Netherlands). Additionally, SEM images were obtained of etched substrates of the respective groups.

Diametral tensile strength of all 4 resin composite cements was measured [55, 57, 61, 62]. Cylindrical test specimens 3 mm in height and diameter (*n* = 10) were produced using a customized Teflon mold. The cement was filled into the respective cavities of the mold and kept in place with a plastic foil and a glass plate on each side. Specimens were light cured for 20s from both sides (Elipar DeepCureS, 3 M Espe). All specimens were then stored in 37 °C water for 24 h. Specimens were loaded until fracture after 24 h of water storage using a universal testing machine (Z020, Zwick/Roell). Cross-head speed was set to 1 mm/min. Prior to the measurements, the specimens were sized in diameter and height using a digital caliper (Cal IP 67, Tesa, Ingersheim, Germany). Diametral tensile strength values were calculated using the following equations:

$$ {\upsigma}_{\mathrm{t}}=2\mathrm{F}/\uppi \mathrm{dh} $$

F is the fracture load; d the specimen diameter and h the specimen height.

All data was tested for normal distribution using Shapiro-Wilk test (StatPlus Pro, v6.1.25, AnalystSoft; Walnut, CA, USA) (*p* < 0.05). To analyze diametral tensile strength one-way ANOVA was applied followed by Fisher LSD test to investigate differences between resin composite cements (p < 0.05). For SBS data one-way ANOVA was performed for each cement to test the influence of etching time. Three-way ANOVA was applied for each etching time to test for effects of the factors substrate, HF concentration, and cement. Post-hoc Fisher LSD test was performed to determine differences within the subgroups (*p* < 0.05).

## Results

**Fig. 2:**
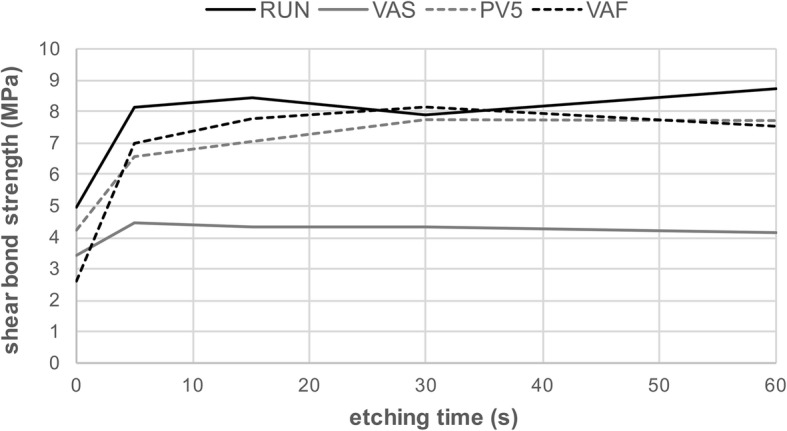
Pooled shear bond strength means for the cements RUN, VAS, PV5 and VAF on all restorative materials using different etching times (0 s, 5 s, 15 s, 30s, 60s) of both HF 5% and HF 9% (*n* = 80 per measuring point)

Shear Bond strength means and standard deviations of all groups with statistics are given in Table 2. Overall, significantly highest values were observed for etching time of 60s = 30s = 15 s ≥ 5 s > 0 s (*p* < 0.001). For the cements, statistically significantly highest SBS values were recorded for RUN>PV5 = VAF > VAS (p < 0.001). Etching with HF5% for 5 s to 15 s resulted in higher SBS values (*p* < 0.005) while no differences were observed between HF5% and HF9% buffered when the substrates were etched for 30s to 60s (*p* > 0.05). The correlation between SBS values and etching time of the pooled data for all materials and HF concentrations is displayed in Fig. 2.

**Table 2:** Shear bond strength means and standard deviations for the different groups (*n* = 10)

shear bond strength mean ± SD (Mpa)			etching time (s)				
substrate	HF concentration	cement	0	5	15	30	60
VM	5%	PV5	6.9 ± 1.2^A^	7.0 ± 1.4^A^	7.6 ± 1.0^A^	8.3 ± 2.1^A^	7.2 ± 0.9^A^
		VAF	3.1 ± 0.4^A^	7.2 ± 1.0^B,C^	7.5 ± 1.5^B,C^	8.0 ± 1.6^B^	6.0 ± 1.3^C^
		RUN	4.7 ± 0.9^A^	8.4 ± 1.1^B^	9.1 ± 1.9^B^	8.0 ± 1.4^B^	8.9 ± 1.0^B^
		VAS	3.2 ± 0.6^A^	5.2 ± 1.0^B^	4.0 ± 0.6^A^	3.9 ± 0.7^A^	3.8 ± 0.7^A^
	9%	PV5	6.9 ± 1.2^A^	7.3 ± 1.5^A^	7.8 ± 0.9^A^	8.0 ± 1.0^A^	7.1 ± 0.9^A^
		VAF	3.1 ± 0.4^A^	7.1 ± 1.4^B^	7.6 ± 1.5^B,C^	8.6 ± 1.6^C^	7.9 ± 1.5^B,C^
		RUN	4.7 ± 0.9^A^	6.5 ± 0.6^B^	7.5 ± 0.6^B,C^	6.6 ± 1.2^B^	7.7 ± 1.5^C^
		VAS	3.2 ± 0.6^A,B^	3.4 ± 0.5^A,B^	3.5 ± 0.6^A,B^	3.9 ± 0.8^A^	2.9 ± 0.7^B^
VE	5%	PV5	5.2 ± 0.8^A^	7.2 ± 1.1^B^	7.7 ± 1.3^B^	7.5 ± 1.2^B^	8.0 ± 1.2^B^
		VAF	4.1 ± 0.9^A^	6.8 ± 0.9^B^	9.0 ± 2.5^C^	9.1 ± 1.9^C^	8.1 ± 1.3^B,C^
		RUN	4.9 ± 0.7^A^	8.1 ± 0.7^B^	8.9 ± 0.6^C^	8.2 ± 0.9^B,C^	7.7 ± 0.8^B^
		VAS	2.3 ± 0.5^A^	4.4 ± 0.9^B^	3.6 ± 0.7^C^	3.7 ± 0.7^B,C^	4.1 ± 0.9^B,C^
	9%	PV5	5.2 ± 0.8^A^	7.1 ± 0.8^B^	7.4 ± 1.1^B^	7.3 ± 1.1^B^	7.5 ± 1.6^B^
		VAF	4.1 ± 0.9^A^	7.2 ± 1.6^B^	8.2 ± 1.5^B^	7.9 ± 1.4^B^	7.6 ± 1.1^B^
		RUN	4.9 ± 0.7^A^	6.5 ± 0.8^B^	7.7 ± 0.8^C^	7.5 ± 0.9^C^	7.4 ± 1.0^C^
		VAS	2.3 ± 0.5^A^	3.4 ± 0.7^B^	3.8 ± 0.6^B,C^	4.2 ± 0.8^C^	4.0 ± 0.7^B,C^
EC	5%	PV5	1.8 ± 1.1^A^	6.1 ± 1.1^B^	6.1 ± 0.9^B^	8.1 ± 1.0^C^	8.6 ± 1.7^C^
		VAF	2.1 ± 1.5^A^	7.2 ± 1.2^B^	7.4 ± 0.9^B^	7.3 ± 0.7^B^	7.8 ± 1.7^B^
		RUN	5.9 ± 2.2^A^	8.3 ± 1.9^B^	8.5 ± 1.5^B,C^	8.1 ± 1.5^B^	10.1 ± 0.8^C^
		VAS	3.7 ± 1.2^A^	5.2 ± 0.9^B^	5.0 ± 1.1^B^	4.9 ± 0.8^B^	4.9 ± 1.3^B^
	9%	PV5	1.8 ± 1.1^A^	4.9 ± 1.3^B^	6.8 ± 1.2^C^	9.5 ± 1.3^D^	8.7 ± 1.3^D^
		VAF	2.1 ± 1.5^A^	6.1 ± 1.4^B^	7.2 ± 0.8^B^	7.0 ± 1.3^B^	7.2 ± 1.4^B^
		RUN	5.9 ± 2.2^A^	9.0 ± 2.2^B^	8.1 ± 2.1^B^	6.8 ± 1.0^B^	9.0 ± 1.3^B^
		VAS	3.7 ± 1.2^A^	4.6 ± 0.6^B^	5.1 ± 0.8^B^	4.5 ± 0.5^A,B^	4.4 ± 0.6^A,B^
VS	5%	PV5	2.9 ± 0.6^A^	7.1 ± 1.4^B^	6.6 ± 1.4^B^	6.8 ± 1.4^B^	7.6 ± 1.4^B^
		VAF	1.2 ± 0.7^A^	6.9 ± 1.5^B^	8.1 ± 1.2^B,C^	9.2 ± 1.8^C^	7.7 ± 1.5^B^
		RUN	4.3 ± 2.0^A^	9.7 ± 0.8^B^	9.3 ± 1.2^B^	9.4 ± 0.6^B^	9.8 ± 1.3^B^
		VAS	4.4 ± 0.8^A^	4.4 ± 1.0^A^	5.2 ± 1.2^A^	4.5 ± 0.8^A^	4.6 ± 1.1^A^
	9%	PV5	2.9 ± 0.6^A^	6.2 ± 1.6^B^	6.2 ± 0.4^B^	6.4 ± 1.2^B^	7.1 ± 1.9^B^
		VAF	1.2 ± 0.7^A^	7.5 ± 1.1^B^	7.5 ± 1.4^B^	8.1 ± 1.7^B^	8.2 ± 1.6^B^
		RUN	4.3 ± 2.0^A^	8.6 ± 1.6^B,C^	8.4 ± 1.1^B,C^	7.8 ± 0.7^B^	9.3 ± 1.7^C^
		VAS	4.4 ± 0.8^A^	5.0 ± 1.0^A^	4.5 ± 1.1^A^	5.1 ± 0.9^A^	4.5 ± 0.9^A^

Statistical differences determined with one-way ANOVA between etching times are indicated with different superscript letters (horizontal comparison, p < 0.05). Statistical differences between substrate, HF concentration and cement type determined with three-way ANOVA within each etching time are provided at the bottom of the table, the ranking starts with the highest mean SBS values (*p* < 0.05)

0 s: VM = VE > EC=VS (*p* < 0.001) / RUN>PV5 > VAS > VAF (*p* < 0.001)

5 s: VS ≥ VM = EC=VE (*p* = 0.007) / RUN>VAF=PV5 > VAS (*p* < 0.001) / HF5 > HF9 (p = 0.005)

15 s: VE = VS=VM = EC (*p* = 0.471) / RUN>VAF > PV5 > VAS (*p* < 0.001) / HF5 > HF9 (*p* = 0.003)

30 s: EC=VS=VE = VM (*p* = 0.803) / VAF = RUN=PV5 > VAS (*p* < 0.001) / HF5 = HF9 (*p* = 0.117)

60 s: EC=VS > VE = VM (*p* < 0.001) / RUN>PV5 = VAF > VAS (*p* < 0.001) / HF5 = HF9 (*p* = 0.057)

**Fig. 3:**
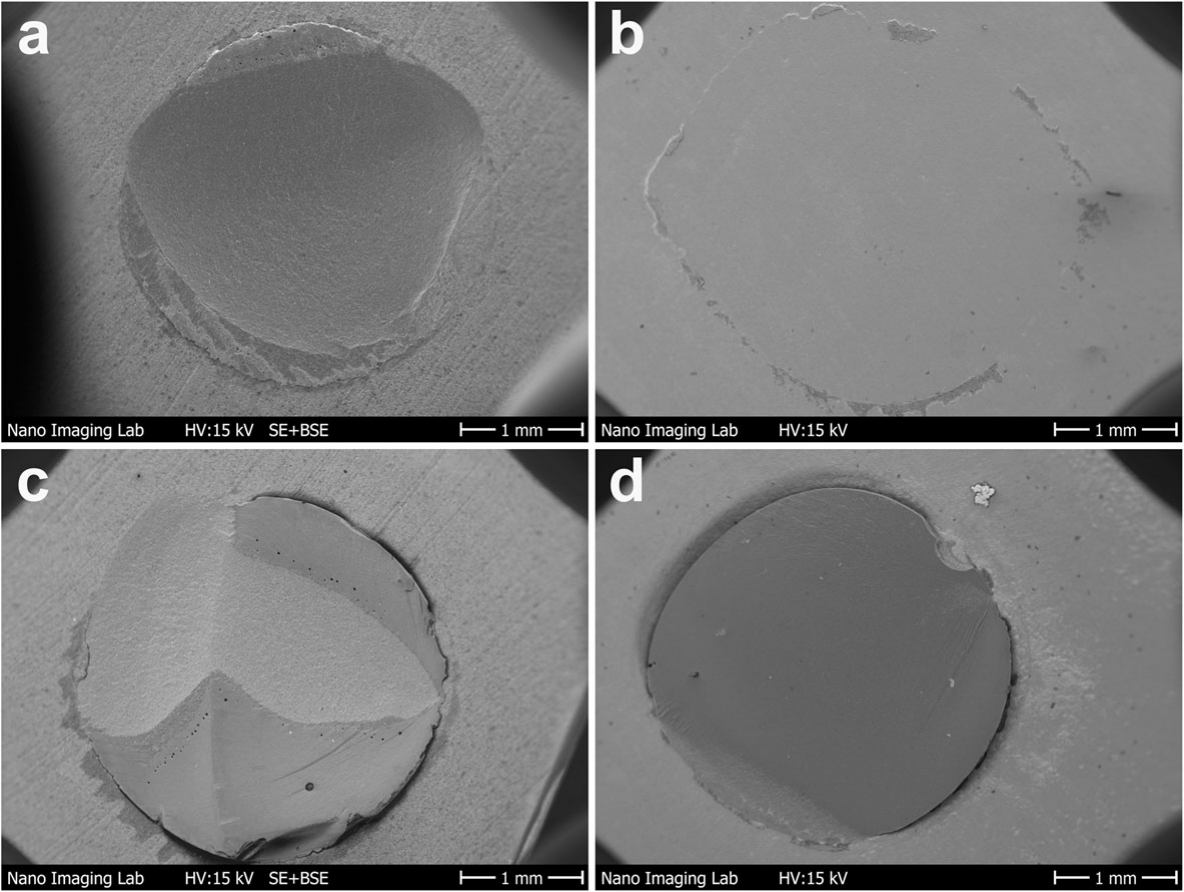
SEM images of typical failure patterns **a**) cohesive failure within the substrate **b**) adhesive failure **c**) mixed failure **d**) cohesive failure within the cement

No etching of the surfaces resulted in adhesive fractures on all substrates. For VAS, fractures were adhesive or within the resin composite cement irrespective of the surface pretreatment on all substrates. For the other cements, fractures of etched surfaces of VM and VE were mainly cohesive in the restorative material or mixed failures and for EC and VS mainly cohesive within the resin composite cement or mixed failures. SEM images of typical failures modes are presented in Fig. 3.

**Fig. 4:**
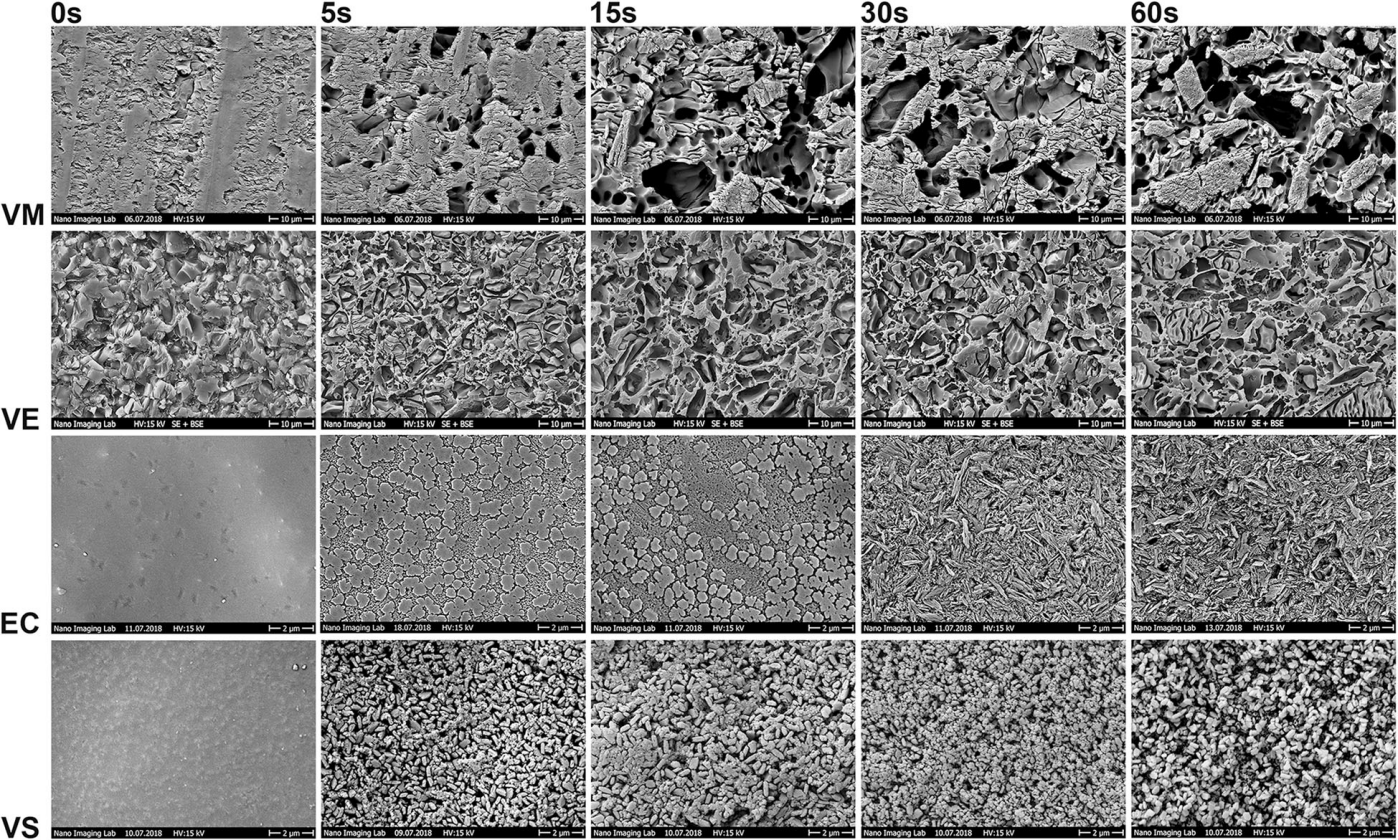
SEM images of substrate surfaces feldspar ceramic (VM), polymer-infiltrated ceramic (VE), lithium disilicate (EC) and zirconia reinforced lithium silicate (VS) pre-treated with 5% hydrofluoric acid for the respective times 0 s, 5 s, 15 s, 30s and 60s. No differences were observed with SEM between HF5 and HF9. Magnification for VM and VE is 2′000x, for EC and VS 10′000x

SEM analysis revealed no differences in etching morphology of the substrates between HF5 and HF9, and images are displayed for HF5 (Fig. 4). Prolonged etching up to 60s, increased the irregularities, undercuts and removal of ceramic particles. HF etching for 5 s or 15 s on EC with either of the concentrations did not suffice to dissolve the glassy matrix completely creating a different pattern than those of 30s to 60s.

Diametral tensile strength of the resin composite cement was significantly higher for PV5 (*p* < 0.005) than for VAF (*p* = 0.005) and RUN (*p* = 0.009) that did not differ significantly (*p* = 0.823) (Table 3). VAS presented significantly lower diametral tensile strength values than all other cements (*p* < 0.001).

**Table 3:** Mean and standard deviation of diametral tensile strength of resin composite cements RUN, VAS, PV5 and VAF (*n* = 10)

	Diametral tensile strength (MPa)
PV5	46.4 ± 3.6^A^
VAF	41.9 ± 2.5^B^
RUN	42.2 ± 4.8^B^
VAS	17.6 ± 1.7^C^

Statistical differences between etching times are indicated with different superscript letters (*p* < 0.05)

## Discussion

The purpose of the present study was to determine the effect of surface pretreatment with 5 and 9% hydrofluoric acid of four different CAD/CAM ceramic materials on shear bond strength of different resin composite cements. Additionally, diametral tensile strengths of four resin composite cements were investigated. It was demonstrated that irrespective of the ceramic material, a minimal etching time of 15 s with 5% or 9% HF is required to achieve the highest shear bond strength. When performing SBS testing with a cement with a low diametral strength, cohesive fractures within the cement and consequently lower SBS values can be expected.

The present study partially rejected the first hypothesis that different HF acid concentrations affect the surface morphology of different CAD/CAM ceramic materials and shear bond strength using four different resin composite cements. The investigated HF acid concentrations of 5 and 9% buffered revealed only differences for etching times of 5 s and 15 s where HF5% displayed higher SBS values. This might be due to a prolonged reaction time of HF9% due to its buffered composition. However, no differences were observed in SEM images. Other findings report significant difference in SBS mean values between HF acid concentrations of 5, 7.5 and 10% for lithium disilicate ceramic [32, 39] and no significance between 5 and 10% HF acid for feldspar and lithium disilicate ceramics were found [34].

The second hypothesis that HF acid etching time affects the substrate’s surface morphology and SBS was confirmed. The highest SBS values for all tested CAD/CAM ceramic materials were observed after etching times of 15 s to 60s, which were accompanied by increased structural irregularities and undercuts due to removal of ceramic particles that are responsible for sufficient micromechanical retention [36, 63]. In the present study differing etching patterns were observed for EC between 15 s and 30s that did not affect SBS values. For practical reasons and to ensure complete etching of the substrate with sufficient dissolution of the glassy matrix the authors recommend an etching time with HF for longer than 15 s and up to 60s as the manufacturer also recommends an etching time of 20s.

Most studies reported the importance of ceramic surface pretreatment with HF acid of 4.8 to 10% and application for 15 s to 60s which agrees with the present findings [40–46, 64]. Recent scientific data determined no significant differences in SBS values regarding prolonged etching time from 20s to 120 s for feldspar and LiS2 ceramic [32, 34]. It is recommended to etch a polymer-infiltrated ceramic for 30s to 60s in order to achieve the highest bond strength [30, 33].

Substrate surface pretreatment without HF etching in the present study served as a control group to assess bonding capacity to machined surface limited in mechanical interlocking. Thus, mainly the chemical bond may have been measured, which resulted in lowest SBS values for all materials and induced mostly adhesive fractures. Even though the machined surfaces were silanized, the fractures for all materials occurred basically on the bonding interface between ceramic surface and cement. These findings are in agreement with previous reports for glass-based ceramics and polymer-infiltrated ceramic [30, 35, 50]. The surface roughness Ra of VM (1.9 ± 0.5 μm), VE (1.8 ± 0.8 μm), EC (0.5 ± 0.1 μm) and VS (0.8 ± 0.1 μm) of the unetched substrates differed, which can be explained by the crystallization process of EC and VS. Therefore SBS values of unetched specimens were also significantly higher of the rougher substrates VM and VE. Reported surface roughness values Ra for VM and VE after CAD/CAM proceeding were found to be Ra = 1.9 μm [60] and it was similar to the values of the present study (VM: 1.9 μm, VE: 1.8 μm). However, EC and VS had lower roughness in the present study (EC: 0.5 μm, VS: 0.8 μm) compared with other reports (EC:2.7 μm, VS:2.5 μm) [60]. It can be explained by the hardness of EC and VS, which might be less susceptible to the treatment with 180 grit silica paper than to the grinding instruments of a CAD/CAM unit.

In the present study, silane application for different cements was strongly followed by manufacturer instructions to avoid chemical interferences. Hence, the primer consisting MDP monomer (10-Methacryloyloxydecyl dihydrogen phosphate) was only applied for PV5, but no superior effect was observed when compared to other primers containing only silane as a bonding agent. Applied on the etched surface, the silane coupling agent creates a chemical bond between Si-O-Si groups in silicate ceramic and methacrylate groups in resin composite cement, and strengthens the adhesion between both materials [30]. Scientific data demonstrated that a silane coupling agent improves the SBS between resin composite cement and silicate ceramic as well as polymer-infiltrated ceramic [33, 49]. A previous study reported slightly higher SBS values sheared with a 2 mm distance for RUN on VE after etching for 15 s to 60s followed by the application of a silane ranging from 9.0 ± 2.9 MPa to 10.1 ± 1.5 MPa, that may be explained by the use of a different silane [30, 33].

The present study revealed mainly cohesive fractures within etched restorative materials of VM and VE with all luting cements except VAS. This indicates that the formed bond between resin composite cement, silane and substrate was stronger than the intrinsic strength of the substrate material itself. EC and VS groups experienced also mainly cohesive fractures, however, within the cement and not the substrate. This observation has been reported previously [25], and is most likely related to the variation in the materials’ flexural strength: VM 105 MPa [14] and VE 137 MPa [14] < EC 348 MPa [15] and VS 443 MPa [15]. The high strength of EC and VS resulted in fractures within the cements. For VM and VE with its increased roughness as shown in SEM images, a strong interlocking between cement and substrates led to cohesive fractures within the restorative material.

In the current study, the specimens were sheared with a 2 mm distance between loading piston and bonding area. Fractures of specimens in a SBS test set-up occur when either the maximal normal stress or shear stress levels are overstepped. When the distance between force application point and fracture area is increased, normal stress is also increased at the same force level. Consequently, fractures occur at lower force levels than when specimens are sheared without distance. SBS values of the present study are therefore lower and cannot be compared to previous studies using the same test set-up [33, 50]. The distance of 2 mm was selected in the present protocol because less crucial cohesive material fractures were observed when specimens were sheared with 2 mm distance due to the increased leverage effect leading to fractures at lower forces [30, 33]. The applied shear bond strength design can be considered a comparable method to the ISO 29022 shear test, although the SBS values obtained with the present design were generally lower than those generated with the ISO test [50]. SBS testing is a valuable method to asses bonding performance between interfaces as long as failures occur at the interface with no fractures of the substrate. As soon as cohesive fractures are involved, the test method has been criticized as unreliable [50, 65, 66].

Further, the low SBS values of VAS led to either adhesive or cohesive fractures within the cement. The diametral tensile strength of 17.6 ± 1.7 MPa was significantly lower than the values of RUN, PV5 and VAF. This could explain the nature of the fractures found with VAS.

The samples in the recent study were evaluated after 24 h water storage at 37 °C without considering the effect of aging. Further investigations regarding bond strength between novel CAD/CAM materials and composite resin cements, as well as regarding surface pretreatment of both tooth and restorative material have to be investigated under clinical conditions.

## Conclusions

Within the limitations of this study the recommended surface pretreatment of VM, VE, EC and VS is HF etching with concentrations of 5% or 9% for 15 s to 60s. Furthermore, the tested resin composite cements can be applied with all tested materials and suggested for clinical application as follow: RUN>PV5 = VAF > VAS.

## Data Availability

Data are available on request from the authors.

## Abbreviations

CAD/CAM: Computer-aided-design/computer-aided-manufacturing
EC: e.max CAD
HF: Hydrofluoric acid
LiS2: Lithium disilicate
PV5: Panavia V5
RUN: RelyX Unicem 2 Automix
SBS: Shear bond strength
VAF: Vita Adiva F-Cem
VAS: Vita Adiva S-Cem
VE: Vita Enamic
VM: Vita Mark II
VS: Vita Suprinity
